# Impaired Relaxation and Reduced Lusitropic Reserve in Atrial Myocardium in the Obese Patients

**DOI:** 10.3389/fcvm.2021.739907

**Published:** 2021-10-27

**Authors:** Yan Wen, Peter M. Deißler, Uwe Primeßnig, Simon Dushe, Volkmar Falk, Abdul Shokor Parwani, Leif-Hendrik Boldt, Florian Blaschke, Christoph Knosalla, Herko Grubitzsch, Burkert M. Pieske, Frank R. Heinzel

**Affiliations:** ^1^Department of Internal Medicine and Cardiology, Charité University Medicine, Campus Virchow-Klinikum, Berlin, Germany; ^2^Department of Intensive Care Unit, The First Affiliated Hospital of Fujian Medical University, Fuzhou, China; ^3^German Center for Cardiovascular Research (DZHK), Partner Site Berlin, Berlin, Germany; ^4^Berlin Institute of Health (BIH), Berlin, Germany; ^5^Department of Cardiovascular Surgery, Charité-Universitätsmedizin Berlin, Corporate Member of Freie Universität Berlin, Humboldt-Universität zu Berlin, Berlin, Germany; ^6^Department of Cardiothoracic and Vascular Surgery, German Heart Institute Berlin, Berlin, Germany; ^7^Department of Cardiothoracic and Vascular Surgery, German Heart Center Berlin, Berlin, Germany; ^8^Department of Internal Medicine and Cardiology, German Heart Center Berlin, Berlin, Germany

**Keywords:** right atrial compliance, obesity, BMI, atrial trabeculae, early subclinical changes

## Abstract

**Background:** Obesity can influence the structure and function of the atrium, but most studies focused on the relationship of body mass index (BMI) and overt left atrium (LA) dysfunction as assessed by clinical imaging. We combined the assessment of right atrium (RA) function *in vivo* and *in vitro* in obese and non-obese patients scheduled for elective cardiac surgery.

**Methods:** Atrial structure and function were quantified pre-operatively by echocardiography. RA tissue removed for the establishment of extracorporeal support was collected and RA trabeculae function was quantified *in vitro* at baseline and with adrenergic stimulation (isoproterenol). Fatty acid-binding protein 3 (FABP3) was quantified in RA tissue. Results were stratified according to the BMI of the patients.

**Results:** About 76 patients were included pre-operatively for the echocardiographic analysis. RA trabeculae function at baseline was finally quantified from 46 patients and RA function in 28 patients was also assessed with isoproterenol. There was no significant correlation between BMI and the parameters of atrial function measured by the clinical echocardiography. However, *in vitro* measurements revealed a significant correlation between BMI and a prolonged relaxation of the atrial myocardium at baseline, which persisted after controlling for the atrial fibrillation and diabetes by the partial correlation analysis. Acceleration of relaxation with isoproterenol was significantly lower in the obese group (BMI ≥ 30 kg/m^2^). As a result, relaxation with adrenergic stimulation in the obese group remained significantly higher compared to the overweight group (25 kg/m^2^ ≤ BMI < 30 kg/m^2^, *p* = 0.027) and normal group (18.5 kg/m^2^ ≤ BMI < 25 kg/m^2^, *p* = 0.036). There were no differences on impacts of the isoproterenol on (systolic) developed force between groups. The expression of FABP3 in the obese group was significantly higher compared to the normal group (*p* = 0.049) and the correlation analysis showed the significant correlations between the level of FABP3 in the RA trabeculae function.

**Conclusion:** A higher BMI is associated with the early subclinical changes of RA myocardial function with the slowed relaxation and reduced adrenergic lusitropy.

## Introduction

Overweight or obese can cause important health repercussions. Obesity has been linked to a variety of cardiovascular disorders ranging from hyperdynamic circulation through the subclinical cardiac structural changes to overt heart failure (1–3). Obesity is one of the most common causes of structural and functional cardiac remodeling that leads to cardiac dysfunction. A greater degree of adiposity is strongly associated with the concentric left ventricular (LV) remodeling in midlife and the cumulative effects of a longer duration of the overall obesity contribute to the concentric remodeling predominantly by increasing LV mass (4).

Obesity can also influence the structure and function of the atrium. Clinically, body mass index (BMI) is a commonly used parameter to define obesity. Prior reports have described that BMI and increasing age were associated with the left atrium (LA) enlargement and LA volumes that were independent of the effects of hypertension and diabetes mellitus type 2 (DM2) (5). Obese patients with paroxysmal atrial fibrillation (AF) were characterized by the impaired LA global longitudinal strain, which is persistent and was accompanied by the segmental dysfunction after the percutaneous pulmonary vein isolation at the 6-month follow-up (6). Recent studies have demonstrated that the right atrium (RA) volumes have been identified as an independent predictor of the adverse events in the patients with heart failure (HF) with reduced ejection fraction (HFrEF) assessed on both the MRI and echocardiography (7, 8). An inverse association between the increased RA volume index (RAVI) and impaired RA emptying fraction (RAEF) has been reported in the patients with pulmonary arterial hypertension assessed by either echocardiography or MRI (9, 10). Both the larger RAVI and lower RAEF were associated with the 12-month HF hospitalizations or death irrespective of age, sex, and prior AF (11). It is unclear whether the alterations in atrial function observed in adipose patients are solely explained by the systemic effects (mechanical load, inflammation). Alternatively, the chronic atrial remodeling *in vivo* may also reflect the intrinsic obesity-related dysfunction of the atrial myocardium.

Fatty acid-binding protein 3 (FABP3) is an intracellular FA carrier, which has been evaluated as a biomarker of atrial maladaptive remodeling in patients with metabolic disease (12, 13). Correlation of the plasma levels and genetic expression of FABP3 with atriomyopathy suggest a relation between FABP3 expression in the atrial tissue and atrial function; however, this has not yet been explored (14–17).

In this study, RA structure and function were assessed by echocardiography, and RA trabeculae function in the patients with normal BMI and the patients with higher BMI (BMI ≥ 25 kg/m^2^) were quantified. In addition, the RA expression of FABP3 in relation to the functional reserve of the atrial myocardium in the patients with normal and increased BMI was also estimated.

## Methods

### Patient Information

All the participants provided a written informed consent upon enrollment. The data and tissue collection were approved by the Ethics Committee of the Charité-Universitätsmedizin Berlin (No. EA2/167/15). The study conformed to the ethical guidelines of the 1975 Declaration of Helsinki. Enrolled patients underwent either the coronary artery bypass graft (CABG) or valve replacement/reconstruction in the Charité-Universitätsmedizin Berlin and the German Heart Center Berlin from July 1 2018 to January 1, 2020. Patients should be ≥18 years old and should be able to provide written informed consent. Patients with the valve replacement or reconstruction predominantly suffered from the aortic valve disease and, therefore, underwent the aortic valve replacement, but the mitral or tricuspid valve replacement surgeries were also performed in the cohort of patients suffering from tricuspid or mitral valve insufficiencies or stenosis. In this study, the clinical exclusion criteria prior to enrollment were as follows: (1) patients with present active tumor, (2) patients with congenital heart disease, and (3) patients with present active infection. The general information on all the subjects was collected including gender, age, diagnosis, medication, and routine laboratory values (laboratory tests were performed within a week before surgery after the admission of the patient). RA tissue was obtained pre-operatively as excess tissue by following the placement of the cannula for the extracorporeal circulation.

### Retrospective Analysis of the Atrial Function by the Echocardiography

Transthoracic echocardiography was performed within 1 week before the procedure by a single experienced investigator by using an Epiq 7G (Phillips Academy, Andover, Massachusetts, USA) with a 2.5-MHz probe in the two-dimensional (2D), M, and Doppler modes. The routine evaluation consisted of the measurements of the heart chamber dimensions and the assessment of valvular function and myocardial contractility. The loops of transthoracic echocardiography were retrieved and analyzed for atrial function by one of the coauthors (UP) experienced in the echocardiography analysis, who was blinded for those general clinical data of the patients including BMI. Speckle-tracking imaging with the LA and RA strain analysis was performed by using EchoPAC Software version 112.0.0 (GE Healthcare, Horten, Norway). The five-wall model for LA segmentation corresponding with a six-segment division in apical two-chamber (2Ch) and four-chamber (4Ch) projections was used during the LA and RA strain analysis (18).

### Right Atrial Appendages Collection

Samples from the RA appendages [(1–2) cm × (0.5–2) cm, *n* = 76, Supplementary Figure 1] were obtained at the onset of the procedure from the cannulation site (RA) and placed in the cardioplegic Tyrode's solution containing: 100 mM sodium chloride (NaCl), 10 mM potassium chloride (KCl), 1.2 mM potassium dihydrogen phosphate (KH_2_PO_4_), 5 mM magnesium sulfate (MgSO_4_), 5 mM 4-Morpholinepropanesulfonic acid, 3-(N-morpholino)propanesulfonic acid (MOPS), 50 mM taurine, 20 mM glucose, and 30 mM 2,3-butanedione monoxime, equilibrated to a pH of 7.4, and immediately transported to the laboratory. From these RA appendages, the RA tissues were aliquoted and stored in −80°C for the extracted proteins and the atrial trabeculae dissected for the function measurements.

### Right Atrial Trabeculae Preparation

Atrial trabeculae (≥3 mm in length) were immediately dissected from the RA appendages with the help of a stereomicroscope and kept at 4°C in the cardioplegic buffer as previously described (19). The RA trabeculae were mounted in the special chambers between the miniature hooks, connected to an isometric force transducer (Myostatin-intact muscle analysis system, MyoTronic UG, Heidelberg, Germany) and superfused with the normal Tyrode's solution [136 mM NaCl, 4 mM KCl, 1 mM magnesium chloride (MgCl_2_), 10 mM N-(2-hydroxyethyl)piperazine-N′-(2-ethanesulfonic acid) (HEPES), 20 mM glucose, and 2.5 mM calcium chloride (CaCl_2_)] at 37°C. The RA trabeculae were electrically paced with the rectangular pulses (5 ms) field stimulation at 1 Hz and then gradually pre stretched to their maximal force of contraction (19, 20). The maximal steady-state twitch force was reached when further stretching did not cause any greater force of contraction (19, 20). The force transducer transmitted the signal for each muscle contraction cycle via an interface to the respective computer, where these contractions were displayed as individual impact analysis and as an overall analysis in the MyoDat program. An electrically initiated developed force of contraction (mN) was measured at baseline in the steady-state (1 Hz) for 10 min. Normalized developed force (mN/mm^2^) was calculated as follows = Developed force (mN)/Cross-sectional area of trabeculae (mm^2^). The RA trabeculae that presented as persistent arrhythmia at baseline were excluded. Thereafter, the force of contraction was measured in the presence of isoproterenol (20 nM) for 10 min (for trabeculae use, see Supplementary Figure 1; for a description of the parameters analyzed in the *in-vitro* functional experiments, see Supplementary Figure 2).

### Enzyme-Linked Immunosorbent Assay

We used the FABP3 ELISA kit (OKEH00897, Aviva Systems Biology Corporation, San Diego, California, USA) to determine the levels of FABP3 in RA tissue by ELISA exactly as the instruction of the manufactures. The optical density (OD) value was measured by the xMark™ microplate absorbance spectrophotometer (Bio-Rad Laboratories, Inc., Hercules, California, USA).

### Statistical Analysis

The statistical analysis was performed by using the IBM SPSS 22.0 software package and the GraphPad Prism version 7.00. The measurement data were expressed as mean ± SD or mean ± SEM. For the multiple groups comparison, a one-way ANOVA or the chi-squared test was used; a one-way ANOVA was followed by the Tukey's test or the least significant difference (LSD) test. For the comparison of the two groups, the Student's *t-*test was used. The Pearson correlation analysis or partial correlation analysis was used to analyze the correlations between the BMI and other parameters. Correlations between FABP3 and the measurements of the RA trabeculae function were analyzed by the Pearson correlation analysis. All the figures were developed by GraphPad Prism version 7.00. A *p* < 0.05 was considered statistically significant.

## Results

### General Characteristics of the Subjects

In this study, the clinical data and RA tissue from 76 patients were prospectively included between July 2018 and January 2020. The patients were stratified into the three groups based on BMI: normal weight (18.5 kg/m^2^ ≤ BMI < 25 kg/m^2^, *n* = 23), overweight (25 kg/m^2^ ≤ BMI < 30 kg/m^2^, *n* = 33), and obese (BMI ≥ 30 kg/m^2^, *n* = 20). There was no significant difference in age, sex distribution, comorbidities, or routine laboratory values between the groups (Table 1).

**Table 1:** Clinical data.

	**BMI (18.5–25)** **(*n* = 23)**	**BMI (25–30)** **(*n* = 33)**	**BMI ≥ 30** **(*n* = 20)**	***F*** **/χ^2^** **value**	***P*** **-value**
Sex (female, %)	8, 34.8	4, 12.1	5, 25.0	4.20	0.12
Age (years)	64.7 ± 15.3	68.1 ± 9.8	68.1 ± 9.8	0.68	0.51
Hb (g/dl)	14.19 ± 1.57	12.55 ± 2.22	18.13 ± 21.99	1.49	0.23
Na^+^ (mmol/l)	139.04 ± 3.47	139.45 ± 3.23	140.60 ± 3.63	1.19	0.31
K^+^ (mmol/l)	4.21 ± 0.65	4.34 ± 0.44	4.24 ± 0.54	0.43	0.65
Creatinine (mg/dl)	1.11 ± 1.182	1.23 ± 0.59	1.57 ± 1.88	0.79	0.46
CRP (mg/l)	6.84 ± 18.13	16.40 ± 34.08	8.46 ± 13.64	1.12	0.33
TSH (uU/L)	1.90 ± 1.42	1.42 ± 0.87	1.60 ± 0.87	1.12	0.33
LVEF (%)	50.5 ± 2.3, *n* = 22	50.0 ± 1.9	51.7 ± 1.8	0.16	0.85
LVEDD (mm)	49.0 ± 2.1, *n* = 21	48.3 ± 1.2, *n* = 32	49.6 ± 1.2, *n* = 18	0.17	0.84
E/É	10.4 ± 1.1, *n* = 9	11.4 ± 1.0, *n* = 12	14.1 ± 1.8, *n* = 9	1.98	0.16
Co-morbidities and medication (*N*, %)					
Atrial fibrillation	5, 21.7	5, 15.2	3, 15.0	0.48	0.79
CAD	16, 69.6	29, 87.9	16, 80.0	2.84	0.24
Hypertension	16, 69.6	26, 78.8	16, 80.0	0.82	0.66
Diabetes	7, 30.4	9, 39.1	2, 10.0	3.26	0.20
Dyslipidemia	8, 34.8	16, 48.5	6, 30.0	2.09	0.35
Medication (*N*, %)					
ACEI/ARB	18, 78.3	19, 57.6	14, 70.0	2.73	0.26
Valsartan + sacubitril	0	0	2, 10.0	5.49	0.06
Spironolacton/eplerenone	2, 8.7	4, 12.1	5, 25.0	2.39	0.30
β-Blockers	14, 60.9	19, 57.6	15, 75.0	1.70	0.43
Statins	17, 73.9	20, 60.6	16, 80.0	2.52	0.28
Diuretic	11, 47.8	11, 33.3	8, 40.0	1.20	0.55
Type of surgery (*N*, %)					
CABG^a^	18, 78.3	21, 63.6	14, 70.0	3.42	0.49
Valve surgery^b^	3, 13.0	6, 18.2	5, 25.0		
CABG+ valve surgery^c^	2, 8.7	6, 18.2	1, 5.0		

*CABG, coronary artery bypass graft; BMI, body mass index; Hb, hemoglobin; CRP, C-reactive protein; TSH, thyroid stimulating hormone; LVEF, left ventricular ejection fraction; LVEDD, left ventricular end diastolic diameter; CAD, coronary artery disease; ACEI/ARB, angiotensin-converting enzyme inhibitor/angiotensin receptor blocker; valve surgery refers to the heart valve replacement or heart valvuloplasty/cardiac valve repair*.

a *Patients only performed CABG*.

b *Patients only performed valve surgery*.

c *Patients performed valve surgery and CABG; values indicate mean ± SD*.

### Correlations Between the Body Mass Index and Atrial Function Measured by the Echocardiography

The LA and RA morphology and function were assessed pre-operatively by the transthoracic echocardiography.

There was no significant difference between the groups in the LA or RA morphology and function (Table 2). Similarly, the Pearson correlation analysis did not show a significant correlation between BMI and the parameters of the atrial function measured by echocardiography.

**Table 2:** Comparisons of the atrial function measured by the echocardiography between the groups based on BMI.

	**BMI (18.5–25)**	**BMI (25–30)**	**BMI ≥ 30**	***F*** **-value**	***P*** **-value**
LA volume (ml)	72.50 ± 23.27 (*n* = 14)	62.17 ± 12.98 (*n* = 29)	73.00 ± 16.15 (*n* = 14)	2.844	0.067
LA diameter (mm)	38.06 ± 4.30 (*n* = 16)	36.71 ± 2.96 (*n* = 31)	38.47 ± 3.28 (*n* = 17)	1.733	0.185
LA strain (%)	19.33 ± 6.17 (*n* = 12)	21.92 ± 5.73 (*n* = 12)	20.00 ± 7.38 (*n* = 6)	0.538	0.590
LA emptying fraction (%)	46.29 ± 13.18 (*n* = 14)	48.74 ± 9.74 (*n* = 27)	45.14 ± 13.07 (*n* = 14)	0.506	0.606
RA area (cm^2^)	16.79 ± 3.64 (*n* = 14)	16.28 ± 4.67 (*n* = 29)	16.79 ± 2.69 (*n* = 14)	0.114	0.893
RA diameter (mm)	33.00 ± 5.04 (*n* = 14)	33.71 ± 5.95 (*n* = 31)	33.59 ± 4.69 (*n* = 17)	0.084	0.919
RA emptying fraction (%)	48.50 ± 11.91 (*n* = 14)	48.88 ± 11.31 (*n* = 25)	47.67 ± 9.63 (*n* = 12)	0.048	0.953
RA strain (%)	36.90 ± 8.32 (*n* = 10)	41.00 ± 4.95 (*n* = 9)	44.25 ± 3.86 (*n* = 4)	2.041	0.156

*BMI, body mass index; LAVI, left atrial volume index; LA, left atrium; RA, right atrium. Values indicate mean ± SD*.

### Impact of Body Mass Index on the Right Atrial Trabeculae Function

**Figure 1:**
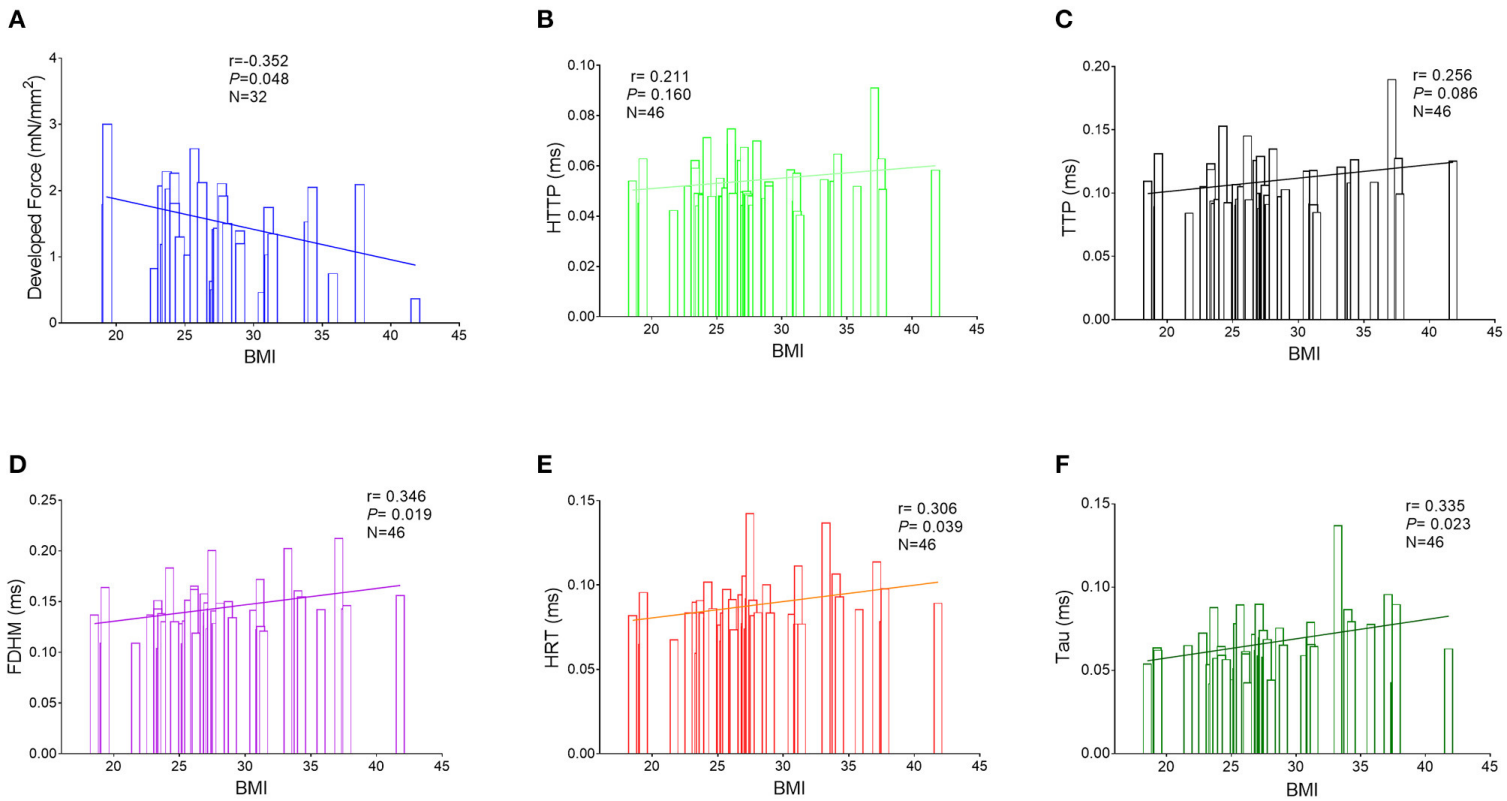
Correlations between the BMI and measurements of the right atrial trabeculae function by the Pearson correlation analysis. **(A)** Correlations between the BMI and normalized developed force (*n* = 32). **(B)** Correlations between BMI and HTTP (*n* = 46). **(C)** Correlations between BMI and TTP (*n* = 46). **(D)** Correlations between BMI and FDHM (*n* = 46). **(E)** Correlations between BMI and HRT (*n* = 46). **(F)** Correlations between BMI and Tau (*n* = 46). BMI, body mass index; HTTP, half time to peak; TTP, time to peak; FDHM, full duration at half maximum; HRT, half relaxation time; Tau, relaxation constant.

**Figure 2:**
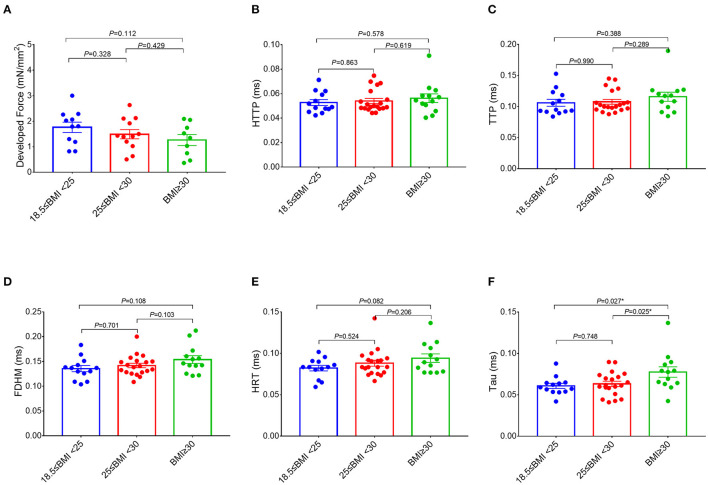
Comparisons of the measurements of the right atrial trabeculae function between the groups. The developed force of the right atrial trabeculae of 32 patients (normal group: *n* = 11; overweight group: *n* = 12; obese group: *n* = 9) was analyzed **(A)** and the rest of the parameters of the right atrial trabeculae of 46 patients (normal group: *n* = 13; overweight group: *n* = 20; obese group: *n* = 13) was analyzed **(B–F)**. Compared with the obese group (BMI ≥ 30 kg/m^2^), Tau in both the overweight group (25 kg/m^2^ ≤ BMI < 30 kg/m^2^) and the normal group (18.5 kg/m^2^ ≤ BMI < 25 kg/m^2^) is significantly lower, but no significant differences on the rest of the parameters of the right atrial trabeculae function between the three groups. ^*^Compared to the obese group (BMI ≥ 30 kg/m^2^), *p* < 0.05. Data are expressed as mean ± SEM. BMI, body mass index; HTTP, half time to peak; TTP, time to peak; FDHM, full duration at half maximum; HRT, half relaxation time; Tau, relaxation time constant.

**Figure 3:**
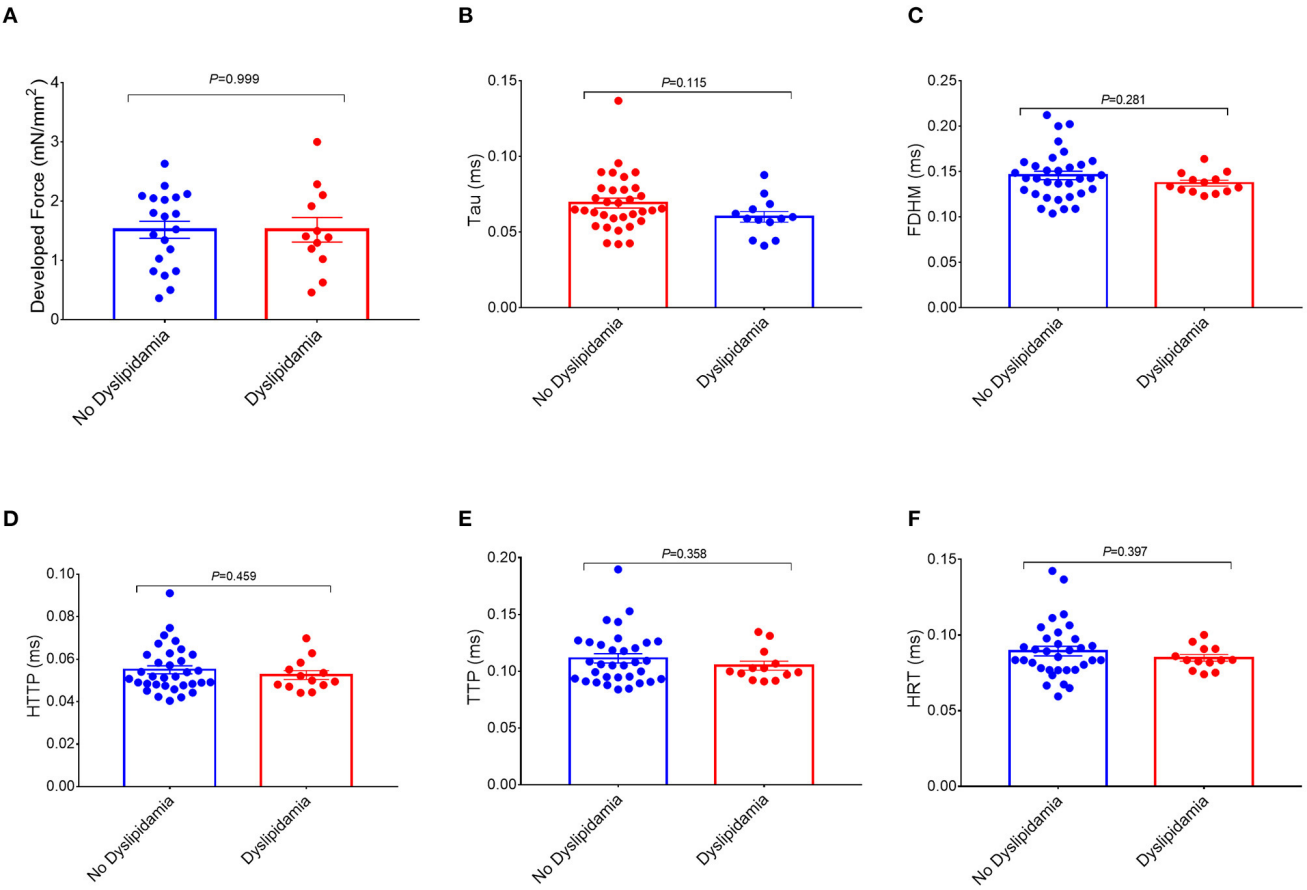
Comparisons of the measurements of the right atrial trabeculae function between the dyslipidemia group and the no dyslipidemia group. The developed force of the right atrial trabeculae of 32 patients (dyslipidemia group: *n* = 12; no dyslipidemia group: *n* = 20) was analyzed **(A)** and the rest of the parameters of the right atrial trabeculae of 46 patients (dyslipidemia group: *n* = 13; no dyslipidemia group: *n* = 33) was analyzed **(B–F)**. There is no significant difference in the measurements of the right atrial trabeculae function between the two groups. Data are expressed as mean ± SEM. BMI, body mass index; HTTP, half time to peak; TTP, time to peak; FDHM, full duration at half maximum; HRT, half relaxation time; Tau, relaxation time constant.

The RA trabeculae from 76 patients were collected and functional analysis was performed. The functional measurements of 30 patients had to be excluded (the exclusion rates in all the three groups are similar) because the RA trabeculae presented persistent arrhythmia at baseline. As a result, RA *in-vitro* function quantified by the RA trabeculae was analyzed in 46 patients (Figures 1, 2). Normalized developed force (Figures 1A, 2A, 3A) is only given for those muscle strips where the cross-sectional area of the muscle strip could be determined as outlined in the methods (*n* = 32). Supplementary Figure 3 shows the absolute developed force in all the muscle strips (*n* = 46) which despite the much larger variation without normalization to the cross-sectional area that confirms a trend toward an association between the developed force at baseline and BMI (Supplementary Figure 3A). There was no significant difference in the age, sex distribution, comorbidities, or routine laboratory values between the groups (Supplementary Table 1) and no significant difference between the groups in the LA or RA morphology or function measured by the echocardiography (Supplementary Table 2).

Interestingly, a higher BMI was associated with slowed relaxation in the RA trabeculae as reflected in a prolonged duration of the contractile cycle [full duration at half maximum (FDHM)] (Figure 1D), a prolonged half time of relaxation (HRT) (Figure 1E), and a significantly higher relaxation constant Tau (the average increase rate of Tau is 3.74% per BMI category, Figure 1F), but only Tau in the obese group showed the significant differences, respectively, compared with the normal group (*p* = 0.027) and the overweight group (*p* = 0.025) (Figure 2F). A trend toward a longer time to the peak contraction was also found (Figures 1B,C). A similar pattern of contractile dysfunction was observed by the partial correlation analysis, controlled for AF and diabetes (Supplementary Table 3). We did not observe any differences in RA myocardial tissue function when the patients were grouped according to the diagnosis of dyslipidemia (Figure 3).

**Figure 4:**
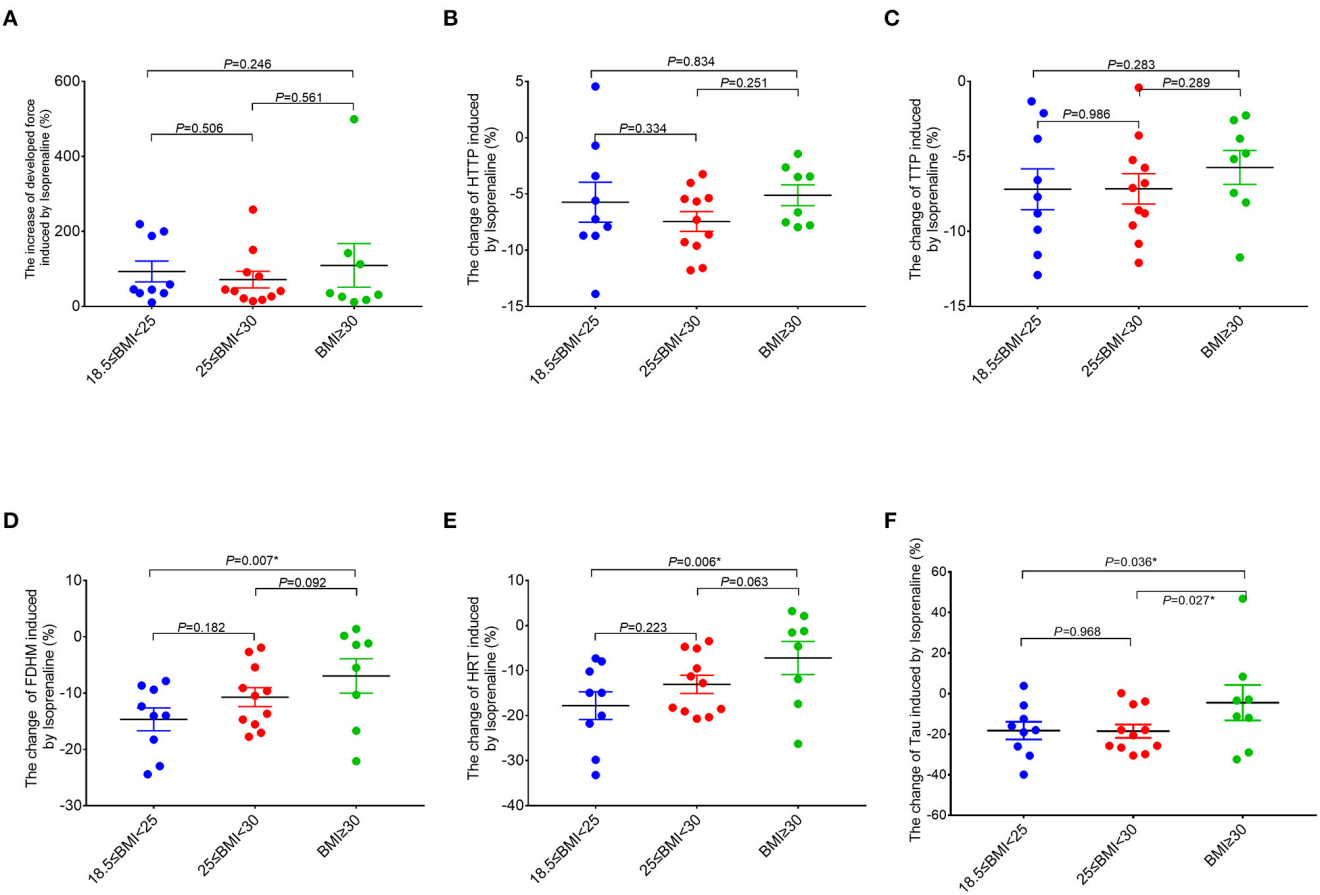
Comparisons of the response of the right atrial trabeculae to isoproterenol between the groups (normal group, *n* = 9; overweight group, *n* = 11; obese group, *n* = 8). In comparison to the normal group (18.5 kg/m^2^ ≤ BMI < 25 kg/m^2^), the ranges of changes of FDHM, HRT, and Tau caused by isoproterenol in the obese group (BMI ≥ 30 kg/m^2^) are significantly smaller (by using the X-axis zero point as the reference line). *Compared to the obese group (BMI ≥ 30 kg/m^2^), *p* < 0.05. All the data are normalized to baseline. Data are expressed as mean ± SEM. BMI, body mass index; HTTP, half time to peak force; TTP, time to peak force; FDHM, full duration at half maximum; HRT, half relaxation time; Tau, relaxation time constant.

In order to further study the effect of the adrenergic stimulation on the RA trabeculae function, inotropic response to the β-adrenergic receptor agonist isoproterenol (20 nM) was studied in all the trabeculae. In 28 patients, those measurements were successfully performed, whereas 18 patients had to be excluded at this point because of the persistent trabeculae arrhythmia after the isoproterenol treatment. The acceleration of FDHM, HRT, and Tau caused by isoproterenol (lusitropy) in the obese group (BMI ≥ 30 kg/m^2^) was significantly smaller compared to the normal group with BMI (18.5 kg/m^2^ ≤ BMI < 25 kg/m^2^) (Figure 4). The Pearson correlation analysis confirmed the significant correlations between BMI and the changes of FDHM and Tau caused by isoproterenol (Supplementary Table 4). No significant differences were observed in the developed force.

### Expression of Fatty Acid-Binding Protein 3 in the Right Atrial Trabeculae and the Relation of FABP3 With the Right Atrial Trabeculae Function and Body Mass Index

**Figure 5:**
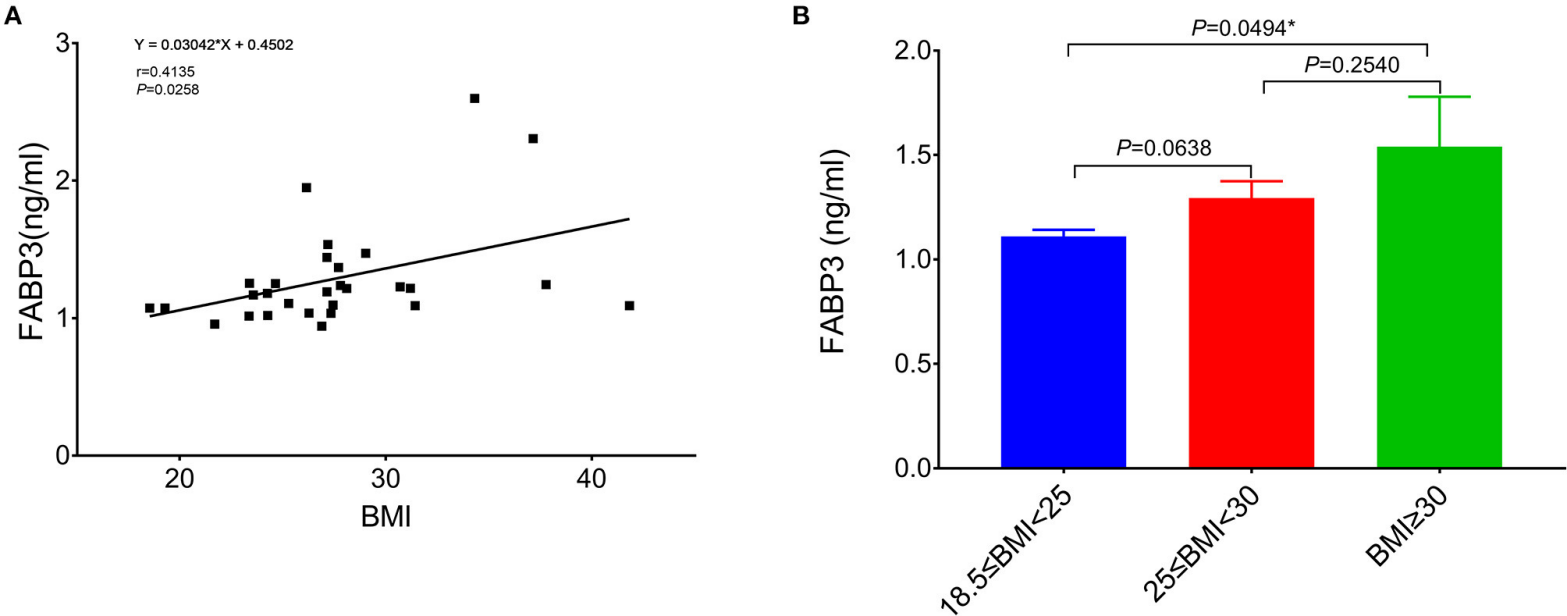
The relation of BMI to FABP3 in the atrium. **(A)** The Pearson correlation analysis shows that the level of FABP3 in the atrium is significantly positively correlated with BMI. **(B)** Comparison of the level of FABP3 in the right atrium between the normal group (18.5 kg/m^2^ ≤ BMI < 25 kg/m^2^, *n* = 10), the overweight group (25 kg/m^2^ ≤ BMI < 30 kg/m^2^, *n* = 12), and the obese group (BMI ≥ 30 kg/m^2^, *n* = 7) and there is a significant difference of the level of FABP3 in the atrium between the normal group and the obese group. *Compared to the obese group, *p* < 0.05. Data are expressed as mean ± SEM. BMI, body mass index; FABP3, fatty acid binding protein 3.

Fatty acid-binding protein 3's expression was analyzed in the RA tissue samples from 29 out of 46 patients (63%) that are included in the final analysis of the RA trabeculae function (other 17 tissues of the patients were not available for analysis). The Pearson correlation analysis shows that the level of FABP3 in the atrium was significantly positively correlated with BMI (Figure 5A). The level of FABP3 in the obese group was significantly higher compared to the normal group (Figure 5B). The Pearson correlation analysis shows significantly positive correlations between the level of FABP3 in RA and the duration of RA myocardial relaxation and there was a trend toward the lower developed force with increased FABP3 (Supplementary Table 5).

## Discussion

In this study, the association of adiposity with RA atrial function in the patients and in the human myocardium *in vitro* had been investigated. For the first time, this study established that, despite unchanged RA function in the clinical echocardiography, a higher BMI was associated with the chronic atrial trabeculae dysfunction as reflected by the impaired relaxation and a decreased lusitropic response of the atrial myocardium. FABP3 may be used as an indicator of the early atrial functional injury caused by higher BMI.

Clinically, increased BMI has been associated with LA enlargement and impaired LA global longitudinal strain (5, 6). We have previously shown that in a rat heart failure model with metabolic syndrome, the LA remodeling and loss of atrial function *in vivo* are direct consequences of the adipose phenotype (21). In this study, no significant correlation between BMI and the LA function was found. This may be explained by the additional factors contributing to LA remodeling such as LV diastolic dysfunction [LV E/e' was in the “gray zone” (i.e., 9–11) of LV diastolic dysfunction in the majority of the patients]. RA volume has been identified as an independent predictor of the adverse events in the patients with HF assessed on both the MRI and echocardiography (7, 8). Larger RAVI and lower RAEF were associated with the 12-month HF hospitalizations or death irrespective of age, sex, and prior AF (11). This study shows the relation of BMI with the RA function measured by echocardiography that did not show a significant correlation. It is important to note that most of the patients selected in this study had no chronic HF and had relatively good left ventricular ejection fraction (LVEF) (68 out of 75 LVEF patients was ≥40%, LVEF value of the one patient was missed). Furthermore, the atrial function (assessed prior to surgery) was still in a compensated phase (which means that there was only mild-to-moderate atrial systolic dysfunction without a decline of the atrial emptying fraction below 37%), 80% of the patients with available LAEF had LAEF ≥37%, and 84.3% of the patients with available RAEF had RAEF ≥37%. Therefore, this study interprets the findings as an early subclinical phase of atrial remodeling.

Through this interpretation, this study established a relationship between the intrinsic atrial trabeculae function and BMI in these patients. The Pearson's correlation analysis showed that BMI correlated with slowed relaxation of the atrial muscle and there were still significantly positive correlations between the BMI and HRT, FDHM, and Tau by the partial correlation analysis, which is independent of AF, diabetes, and not affected by dyslipidemia. The results indicate that a higher BMI may independently cause chronic damage to the atrial trabeculae to function especially on the diastolic function.

Isoproterenol is a non-selective β-adrenoreceptor agonist and the isopropylamine analog of epinephrine (adrenaline). Isoprenaline has positive inotropic, lusitropic (relaxation), and chronotropic effects on the heart. The response of the RA trabeculae to isoproterenol reflects the contractile functional reserve of the atrial trabeculae. Compared to the patients with normal BMI, the increase of developed force induced by isoproterenol in the obese group was not different between the groups indicating the similar inotropy and inotropic contractile reserve. However, the relaxation time and relaxation time constant were longer. This indicates that the adrenergic stimulation does not improve impaired relaxation in the atrial myocardium of these obese patients, which may suggest a structural rather than a functional cause of the contractile dysfunction.

Since many recent studies have shown a significant correlation between BMI and atrial remodeling in recent years (4–6), underlying the overweight- and obesity-related molecular biology mechanisms are yet poorly understood.

In this study, we did not observe any differences in RA myocardial tissue function when the patients were grouped according to the diagnosis of dyslipidemia (Figure 3), which may imply that the circulating lipid abnormalities may not be the only mechanism of obesity-related RA dysfunction. This study proposed that the toxic effect of (by)products of lipid metabolism contributes to those atrial impairments on a local metabolic level.

When the influx of FAs in the cardiomyocytes exceeds the intracellular FA oxidation, FA sequestration as triacylglycerols in the lipid droplets, toxic FA metabolites such as ceramides, diacylglycerols, long-chain acyl-CoAs, and acylcarnitines can accumulate in the cardiomyocytes and cause cardiomyopathy (22). FABP3 is an intracellular lipid-binding protein involved in the cellular FA uptake and intracellular FA transport (12, 13). Higher tissue levels of FABP3 contribute to an increase in the transportation of FA and other lipophilic substances from the cytoplasm to the nucleus.

Most (but not all) studies investigating the relationship between FABP3 and atrial remodeling have shown evidence for a contribution of FABP3 to the atrial remodeling and AF based on FABP3 plasma levels or tissue messenger RNA (mRNA) (14–17). From a mechanistic perspective, the protein concentration of FABP3 in the RA may be better suited to reflect the correlation between FABP3 regulation and RA function. In this study, RA protein expression of FABP3 was investigated and a BMI-dependent increase in the level of RA tissue FABP3 was found. Furthermore, higher levels of FABP3 in RA tissue were related to a slower relaxation of the RA trabeculae function. These results suggest that FABP3 may be used as a mechanistic biomarker and the potential therapeutic target in the atrial metabolic dysregulation with subsequent atrial remodeling and/or chronic atrial dysfunction related to obesity. Based on the earlier studies, the potential mechanisms might be as follows: (1) increased levels of FABP may promote FA accumulation in the RA cells, which then exceeds the oxidative capacity of the cell and induces cardiac lipotoxicity and cardiomyopathy by the peroxisome proliferator-activated receptor signaling pathway (16) and (2) overexpressed FABP3 could result in a marked reduction in cellular ATP production and increase in the production of reactive oxygen species (ROS), eventually causing the mitochondrial dysfunction and damage or upregulate the phosphorylation of mitogen-activated protein kinase (MAPK) signaling pathways and downregulate the phosphorylation of Akt, thus promoting apoptosis (23). This study suggests to further examine the cellular mechanisms of the relationship between higher BMI, atrial FABP3 expression, and atrial dysfunction.

## Limitations

(1) This study investigates the tissue samples from the patients who underwent open-heart surgery, mainly for coronary artery bypass surgery. However, only a minority of the patients had ischemic cardiomyopathy (with LV reduced ejection fraction) and the listed comorbidities reflect a variety of cardiovascular risk factors. Correlations between obesity and atrial function are, therefore, likely found also in the patients with non-coronary artery disease (CAD), but this requires validation. (2) Since this study investigates the tissue samples from the patients that underwent the open heart surgery, routine pre-operative workup did not include the cardiac MRI with the functional imaging techniques. Therefore, the functional *in vivo* analysis of the atrial function only relied on the echocardiographic assessment and could not be supported by the MRI-based strain analysis. However, a good intermodality correlation has been reported for the functional atrial measurements between the transthoracic echocardiography (TTE) and MRI (24). (3) The small atrial tissue samples obtained were used for the functional and protein level analysis, using up most of the tissue available from each patient. Future studies including MRI and histological analyses may provide additional insight into the correlations between atrial function, fibrosis, and obesity. (4) While this study supports the hypothesis that increased FABP3 may be involved in atrial contractile dysfunction and provides evidence on a functional and molecular biology basis, further studies are needed to establish a causal relationship.

## Conclusion

A higher BMI is associated with the early subclinical changes in RA myocardial function with the slowed relaxation and reduced adrenergic lusitropy. FABP3 may be used as an early indicator of atrial dysfunction related to higher BMI.

## Data Availability Statement

The original contributions presented in the study are included in the article/Supplementary Material, further inquiries can be directed to the corresponding author.

## Ethics Statement

The studies involving human participants were reviewed and approved by the Ethics Committee of the Charité-Universitätsmedizin Berlin (No. EA2/167/15). The patients/participants provided their written informed consent to participate in this study.

## Author Contributions

YW collected the patients' clinical data, performed ELISA, analyzed and interpreted all data, and was a major contributor in writing the manuscript. PD analyzed right atrial trabeculae function by Myostation-Intact muscle analysis system. UP did analysis of atrial function by echocardiography. CK, VF, HG, AP, FB, and L-HB collected right atrial appendages from all patients. BP contributed to the design of the study. FH contributed to the design of the study, performed administrative tasks, acquired funding, interpreted the data, and revised the manuscript. All authors contributed to the article and approved the submitted version.

## Funding

This study was supported by the DZHK (German Centre for Cardiovascular Research) site project Multiscale Mechanistic Phenotyping in Heart Failure with Reduced Ejection Fraction (TYPE-HF II, project number 81Z0100204).

## Conflict of Interest

The authors declare that the research was conducted in the absence of any commercial or financial relationships that could be construed as a potential conflict of interest.

## Publisher's Note

All claims expressed in this article are solely those of the authors and do not necessarily represent those of their affiliated organizations, or those of the publisher, the editors and the reviewers. Any product that may be evaluated in this article, or claim that may be made by its manufacturer, is not guaranteed or endorsed by the publisher.
