# Supplementary material for: Impaired Relaxation and Reduced Lusitropic Reserve in Atrial Myocardium in the Obese Patients
Source: Front Cardiovasc Med. 2021 Oct 27;8:739907. doi: 10.3389/fcvm.2021.739907 (PMC8578394; doi:10.3389/fcvm.2021.739907)
Supplement: Supplementary file 1 [file Data_Sheet_1.docx]

**Fig. S1 Flow chart.**

**
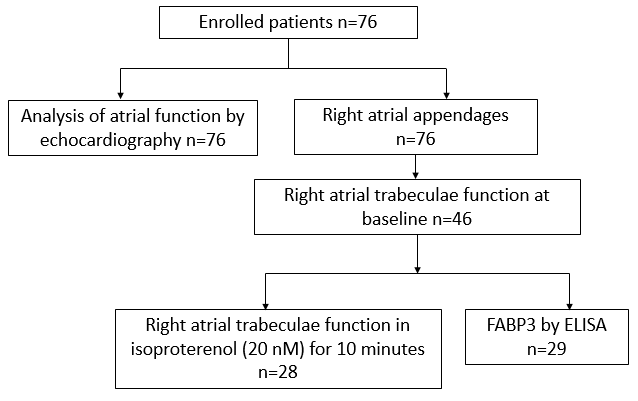
**

**Fig. S2**


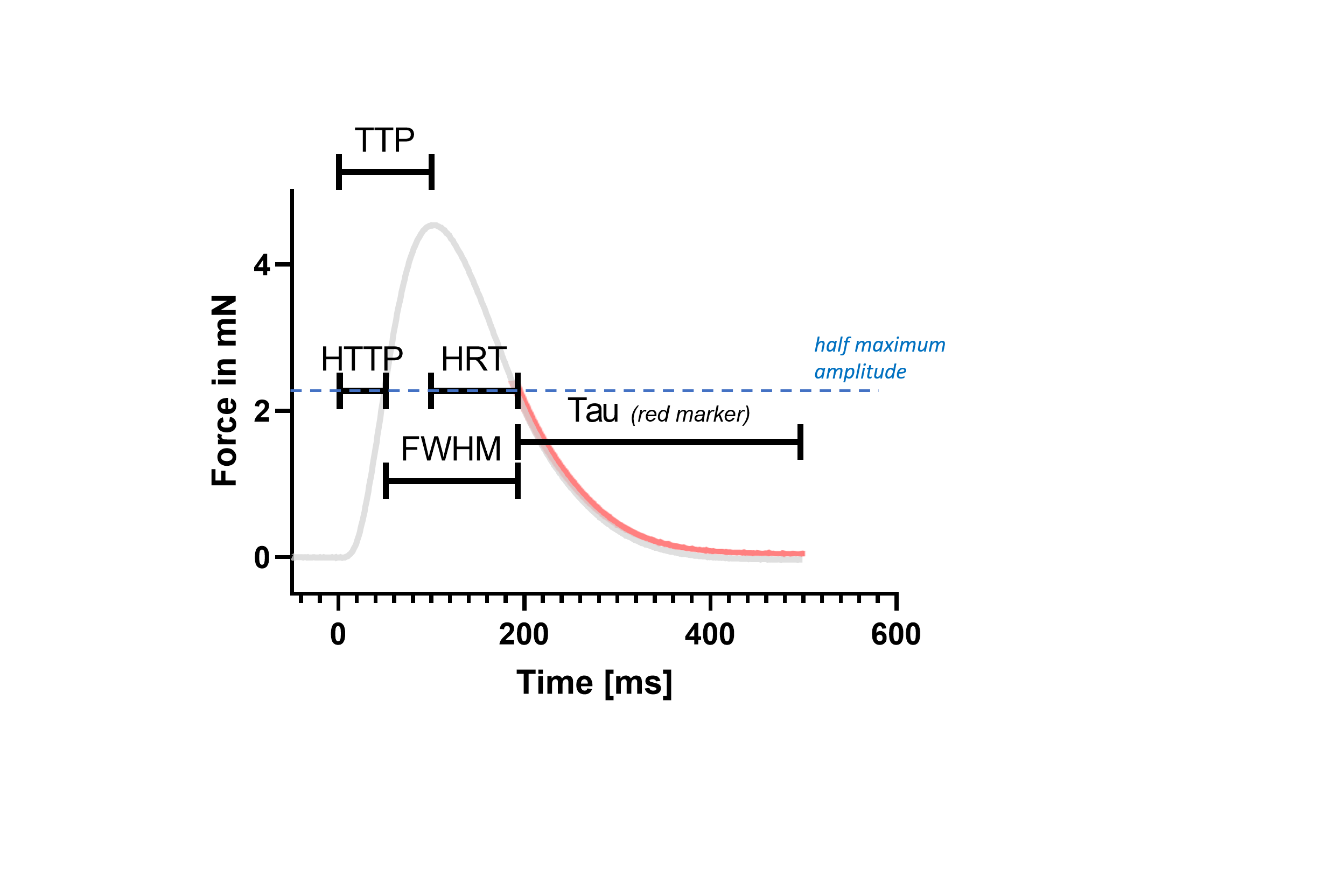


**Fig. S2 Description of parameters analyzed in in-vitro functional experiments.** HTTP: half time to peak; TTP: time to peak; FDHM: full duration at half maximum; HRT: half relaxation time; Tau: relaxation constant. Normalized developed force (mN/mm^2^)= Developed force (mN)/Cross-sectional area of trabeculae (mm^2^).

**Fig. S3**


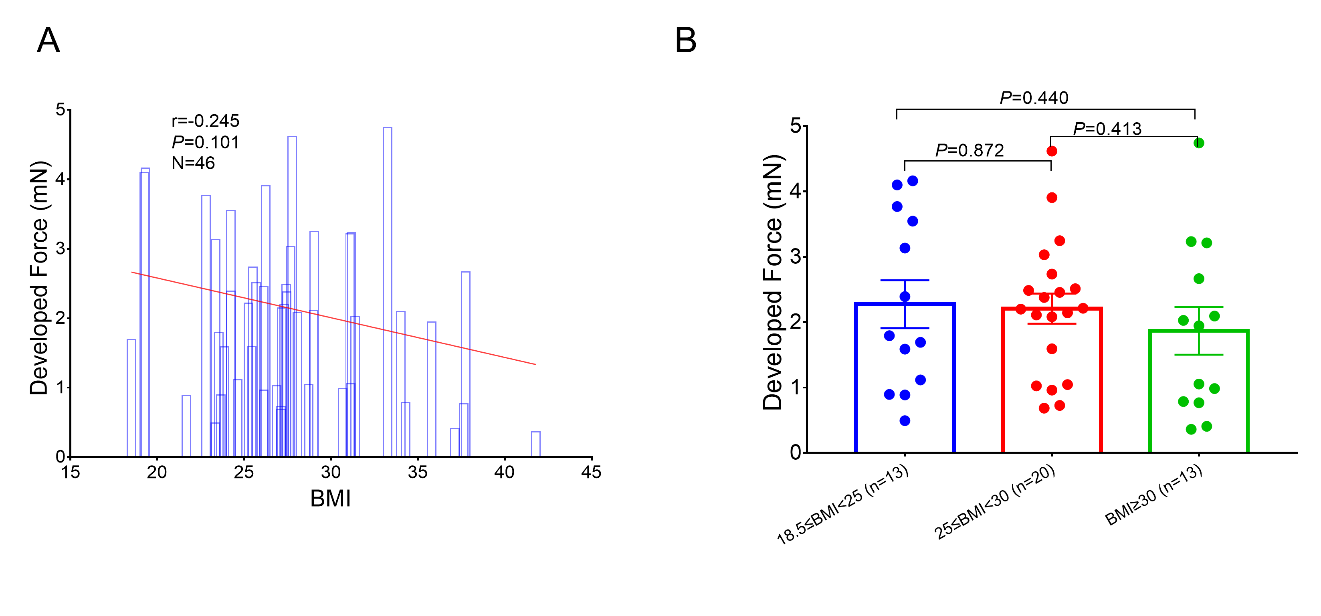


**Fig. S3 Correlation between BMI and developed force (mN) of right atrial trabeculae and comparisons of developed force (mN) of right atrial trabeculae between groups.**

**Table S1 Clinical data of patients selected in final analysis of right atrial trabeculae function.**

|  | BMI (18.5-25) (n=13) | BMI (25-30) (n=20) | BMI≥30 (n=13) | F/ χ^2^ value | *P* value |
| --- | --- | --- | --- | --- | --- |
| Sex (Female, %) | 3, 23.1 | 3, 15 | 2,15.4 | 0.39 | 0.82 |
| Age (Years) | 60.54±18.51 | 69.65±10.61 | 68.08±9.133 | 2.05 | 0.14 |
| Hb (g/dl) | 14.55±1.35 | 12.21±2.32 | 13.33±2.07 | 5.23 | 0.01 |
| Na+ (mmol/l) | 139.92±3.93 | 139.78±3.06 | 141.31±3.68 | 0.81 | 0.45 |
| K+ (mmol/l) | 4.13±0.42 | 4.43±0.45 | 4.29±0.59 | 1.41 | 0.26 |
| Creatinine (mg/dl) | 0.83±0.14 | 1.3±0.48 | 1.92±2.28 | 2.46 | 0.10 |
| CRP (mg/l) | 2.68±3.45 | 29.08±43.36 | 5.13±6.03 | 4.09 | 0.02 |
| TSH (uU/L) | 1.8±1.74 | 1.5±1.05 | 1.52±0.94 | 0.25 | 0.78 |
| LVEF (%) | 52.62±12.31 | 51.5±11.65 | 52.85±8.73 | 0.07 | 0.93 |
| LVEDD (mm) | 48.85±8.34 | 48.7±7.41 | 49.67±5.33 | 0.07 | 0.93 |
| E/É | 9.71±3.15 | 11.7±3.65 | 13.63±5.53 | 1.60 | 0.23 |
| Co-Morbidities and Medication (N, %) | | | | | |
| Atrial Fibrillation | 2, 15.4 | 4, 20.0 | 3, 23.1 | 0.25 | 0.88 |
| CAD | 10, 76.9 | 19, 95.0 | 10, 76.9 | 3.20 | 0.20 |
| Hypertension | 8, 61.5 | 17, 85.0 | 11, 84.6 | 2.78 | 0.25 |
| Diabetes | 4, 30.8 | 6, 30.0 | 1, 7.7 | 3.07 | 0.22 |
| Dyslipidemia | 3, 23.1 | 9, 45.0 | 1, 7.7 | 6.16 | 0.05 |
| Medication (N, %) | | | | | |
| ACEI/ARB | 9, 69.2 | 11, 55.0 | 8, 61.5 | 0.67 | 0.71 |
| Valsartan+Sacubitril | 0 | 0 | 1, 7.7 | 0.26 | 0.28 |
| Spironolacton/Eplerenone | 0 | 0 | 2, 15.4 | 52.9 | 0.07 |
| β-Blockers | 9, 69.2 | 13, 65.0 | 10, 76.9 | 0.54 | 0.76 |
| Statins | 10, 76.9 | 13, 65.0 | 11, 84.6 | 1.70 | 0.43 |
| Diuretic | 7, 53.8 | 5, 25.0 | 5, 38.5 | 2.84 | 0.24 |
| Type of surgery (N, %) |  |  |  |  |  |
| Only CABG | 10, 76.9 | 10, 50.0 | 9, 69.2 | 3.64 | 0.46 |
| Only Valve Surgery | 2, 15.4 | 5, 25.0 | 3, 23.1 |  |  |
| CABG+ Valve Surgery | 1, 7.7 | 5, 25.0 | 1, 7.7 |  |  |

BMI: body mass index; Hb: hemoglobin; CRP: C-reactive protein; TSH: thyroid stimulating hormone; LVEF: left ventricular ejection fraction; LVEDD: left ventricular end diastolic diameter; CAD: coronary atherosclerotic heart disease; ACEI/ARB: angiotensin-converting enzyme inhibitor / angiotensin receptor blocker; valve surgery refers to heart valve replacement or heart valvuloplasty/cardiac valve repair; values indicate mean ± SD.

**Table S2 Comparisons of atrial function measured by echocardiography between groups of patients selected in final analysis of right atrial trabeculae function.**

|  | BMI (18.5-25) | BMI (25-30) | BMI≥30 | F value | *P* value |
| --- | --- | --- | --- | --- | --- |
| LA volume (ml) | 68.22±27.4  (n=9) | 64.79±14.18 (n=19) | 67.30±14.01 (n=10) | 0.133 | 0.876 |
| LA diameter (mm) | 36.90±4.46  (n=10) | 37.05±3.36 (n=20) | 37.50±2.65  (n=12) | 0.095 | 0.910 |
| LA strain (%) | 21.43±6.91 (n=7) | 22.20±6.29 (n=10) | 24.00±8.66  (n=3) | 0.149 | 0.863 |
| LA emptying fraction (%) | 49.67±13.72 (n=9) | 49.67±13.72 (n=19) | 50.40±11.13  (n=10) | 0.103 | 0.902 |
| RA area (cm^2^) | 15.67±3.64 (n=9) | 16.53±5.58 (n=19) | 16.60±2.46  (n=10) | 0.131 | 0.877 |
| RA diameter (mm) | 31.00±3.84 (n=9) | 33.60±6.72 (n=20) | 33.25±4.88  (n=12) | 0.672 | 0.517 |
| RA emptying fraction (%) | 51.11±10.20 (n=9) | 47.89±12.9 (n=18) | 46.88±10.45  (n=8) | 0.322 | 0.727 |
| RA strain (%) | 38.60±9.99 (n=5) | 40.86±5.34 (n=7) | 43.33±4.16  (n=3) | 0.425 | 0.663 |

BMI: body mass index; LAVI: left atrial volume index; LA: Left atrium; RA: right atrium. Values indicate mean ± SD.

**Table S3 Partial correlations between BMI and measurements of right atrial trabeculae function by correlation analysis.**

|  | r value | *P* value | n |
| --- | --- | --- | --- |
| Partial correlation analysis (control variable: atrial fibrillation, diabetes) | | | |
| BMI vs. Developed Force (mN/mm^2^) | -0.335 | 0.070 | 32 |
| BMI vs. HTTP (ms) | 0.222 | 0.147 | 46 |
| BMI vs. TTP (ms) | 0.262 | 0.086 | 46 |
| BMI vs. FDHM (ms) | 0.341 | 0.024* | 46 |
| BMI vs. HRT (ms) | 0.307 | 0.043* | 46 |
| BMI vs. Tau (ms) | 0.354 | 0.018* | 46 |

BMI: body mass index; HTTP: half time to peak; TTP: time to peak; FDHM: full duration at half maximum; HRT: half relaxation time; Tau: relaxation time constant. *: *P*<0.05.

**Table S4 Correlations between BMI and the response of right atrial trabeculae to Isoproterenol by Pearson analysis.**

|  | Pearson r | 95% confidence interval | *P* value | n |
| --- | --- | --- | --- | --- |
| BMI vs. The change of developed force (%) | -0.243 | -0.565 to 0.144 | 0.214 | 28 |
| BMI vs. The change of HTTP (%) | 0.023 | -0.353 to 0.393 | 0.909 | 28 |
| BMI vs. The change of TTP (%) | 0.225 | -0.162 to 0.552 | 0.250 | 28 |
| BMI vs. The change of FDHM (%) | 0.388 | 0.017 to 0.665 | 0.041* | 28 |
| BMI vs. The change of HRT (%) | 0.371 | -0.002 to 0.654 | 0.052 | 28 |
| BMI vs. The change of Tau (%) | 0.529 | 0.195 to 0.754 | 0.004* | 28 |

BMI: body mass index; HTTP: half time to peak; TTP: time to peak; FDHM: full duration at half maximum; HRT: half relaxation time; Tau: relaxation time constant. *: *P*<0.05.

**Table S5 Correlations between FABP3 and the measurements of right atrial trabeculae function by Pearson analysis.**

|  | r value | *P* value | n |
| --- | --- | --- | --- |
| FABP3 (ng/ml) vs. Developed Force (mN/mm^2^) | -0.159 | 0.480 | 22 |
| FABP3 (ng/ml) vs. HTTP (ms) | 0.485 | 0.008* | 29 |
| FABP3 (ng/ml) vs. TTP (ms) | 0.475 | 0.009* | 29 |
| FABP3 (ng/ml) vs. FDHM (ms) | 0.510 | 0.005* | 29 |
| FABP3 (ng/ml) vs. HRT (ms) | 0.469 | 0.010* | 29 |
| FABP3 (ng/ml) vs. Tau (ms) | 0.459 | 0.012* | 29 |

FABP3: cardiac fatty acid binding protein 3; HTTP: half time to peak; TTP: time to peak; FDHM: full duration at half maximum; HRT: half relaxation time; Tau: relaxation constant. *: *P*<0.05.
